# Prioritizing Roadway Pavement Marking Maintenance Using Lane Keep Assist Sensor Data

**DOI:** 10.3390/s21186014

**Published:** 2021-09-08

**Authors:** Justin A. Mahlberg, Rahul Suryakant Sakhare, Howell Li, Jijo K. Mathew, Darcy M. Bullock, Gopi C. Surnilla

**Affiliations:** 1 Joint Transportation Research Program, College of Engineering, Lyles School of Civil Engineering, Purdue University, West Lafayette, IN 47907, USA; jmahlber@purdue.edu (J.A.M.); rsakhare@purdue.edu (R.S.S.); howell-li@purdue.edu (H.L.); kjijo@purdue.edu (J.K.M.); 2 Ford Motor Company, MD 3135, 2101 Village Road Dearborn, Dearborn, MI 48121, USA; gsurnill@ford.com

**Keywords:** pavement markings, lane detection, road maintenance, lane keep assist, LKA, advanced driver assistance systems, ADAS, connected and autonomous vehicles

## Abstract

There are over four million miles of roads in the United States, and the prioritization of locations to perform maintenance activities typically relies on human inspection or semi-automated dedicated vehicles. Pavement markings are used to delineate the boundaries of the lane the vehicle is driving within. These markings are also used by original equipment manufacturers (OEM) for implementing advanced safety features such as lane keep assist (LKA) and eventually autonomous operation. However, pavement markings deteriorate over time due to the fact of weather and wear from tires and snowplow operations. Furthermore, their performance varies depending upon lighting (day/night) as well as surface conditions (wet/dry). This paper presents a case study in Indiana where over 5000 miles of interstate were driven and LKA was used to classify pavement markings. Longitudinal comparisons between 2020 and 2021 showed that the percentage of lanes with both lines detected increased from 80.2% to 92.3%. This information can be used for various applications such as developing or updating standards for pavement marking materials (infrastructure), quantifying performance measures that can be used by automotive OEMs to warn drivers of potential problems with identifying pavement markings, and prioritizing agency pavement marking maintenance activities.

## 1. Motivation

Difficulty in detecting pavement markings can increase driver workload and cause driver confusion, particularly during more challenging driving conditions such as nighttime and/or inclement weather. Vehicles with lane marking sensors and/or autonomous driving encounter similar challenges. Determining locations where vehicles cannot detect pavement markings is especially important in the new frontier of connected and autonomous vehicles. According to a study conducted by the National Cooperative Highway Research Program (NCHRP), approximately 30% of state agencies perform pavement marking evaluations annually, and the remaining agencies collect pavement marking conditions bi-annually or sporadically [1]. Due to the fact of these widely varying evaluation practices and maintenance schedules, this paper proposes using on-board connected vehicle sensors to provide scalable crowdsourced data that will allow agencies to systematically evaluate their road markings and routinely program their maintenance activities.

## 2. Literature Review

A large proportion of crashes (approximately 94%) are caused by human error [2]. This has encouraged many automotive manufacturers to equip advanced driver assistance systems (ADAS) to reduce human error crashes. Original equipment manufacturers (OEM) have equipped lane keep assist (LKA) technology in vehicles over the last decade. The technology’s primary purpose is to enhance safety and comfort for customers, but this technology also enables OEMs to develop algorithms that build up to higher levels of automation [3,4]. Not only does LKA technology enhance user safety, but there is also the potential to provide agencies and drivers feedback on pavement marking quality [5]. The use of LKA provides an efficient and economical opportunity to assess pavement marking quality.

**Figure 1:**
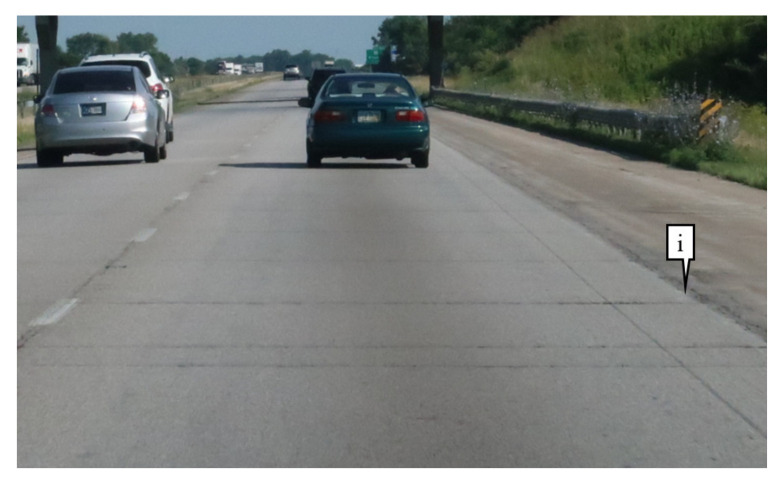
Example of deteriorated pavement marking on roadways.

Pavement markings are used on roads to communicate lane delineations. Lane delineations that are difficult to detect by a vehicle can often confuse drivers and ADAS. Callout i on Figure 1 shows a location where the pavement marking is worn and may be challenging for motorists and autonomous vehicles to delineate between the travel lane and the shoulder.

Identifying locations of low pavement marking quality is particularly important for future connected and autonomous vehicles. Some ADAS technologies, like LKA and lane departure warning (LDW), have not reached the expected market penetration and, even when equipped, may not be utilized [6]. One of the reasons reported for this lack of penetration and use is poor consistency in detecting aged pavement markings [7]. The deterioration of pavement markings is often caused by weather, tire wear, and snowplow wear.

Pavement marking visibility improvements have the potential to reduce crashes and have been reported to reduce wet-road crashes [8]. Previous studies, conducted using a before and after analysis, concluded that the improvement in pavement marking visibility reduced the number of crash incidents, and with the aid of LDW systems, the number of crashes was further reduced [9,10,11]. Another study found that pavement marking retroreflectivity for white edge lines and yellow edge lines was significantly related to crash frequency on four-lane roads [12].

### 2.1. Existing Pavement Marking Metrics and Pavement Markings

Pavement marking evaluation frequency varies by agency, but most agencies use the American Society for Testing and Materials (ASTM) Standard D7585 and ASTM Standard E1710 depending on the instrument being used to evaluate the pavement marking [13,14]. The mobile unit enables agencies to evaluate more pavement markings efficiently, but the unit only detects retroreflectivity on one pavement marking at a time. The unit needs to make three passes to evaluate a two-lane undivided highway.

**Figure 2:**
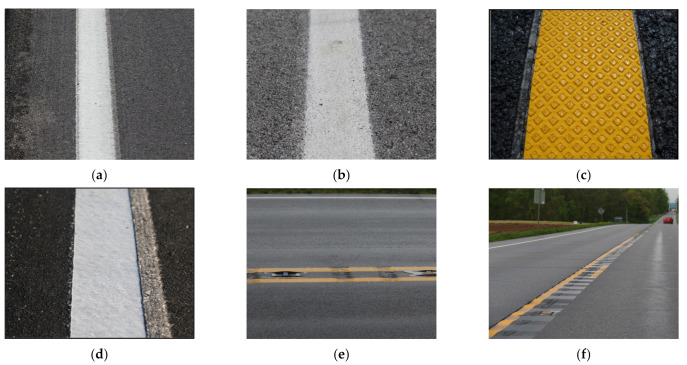
Unique pavement markings: (**a**) multi-component; (**b**) paint; (**c**) preformed tape; (**d**) thermoplastic; (**e**) dry rumble strips; (**f**) wet rumble strips. Image Source: Zehr, S.; Hardin, B.; Lowther, H.; Plattner, D.; Wells, T.; Habib, A.; Bullock, D.M. *Rumble Stripes and Pavement Marking Delineation*; Purdue University: West Lafayette, IN, USA, 2019; doi:10.5703/1288284316937.

Figure 2 shows common types of pavement markings utilized by agencies. The authors of *Rumble Stripes and Pavement Marking Delineation* further provide an in-depth analysis of the quality and longevity of these pavement markings [15]. Preformed tape (Figure 2c) typically has the highest retroreflectivity among the markings shown in Figure 2 [15]. These unique markings show the complexity of environments LKA systems must accommodate and does not account for distressed pavement or other types of pavements such as concrete, brick, or cobblestone. To maximize LKA and LDW safety benefits, inventorying locations where ADAS technologies have detection issues could be used to provide proactive feedback that informs agency maintenance decisions.

### 2.2. Importance of Pavement Markings for Autonomous Vehicles

**Figure 3:**
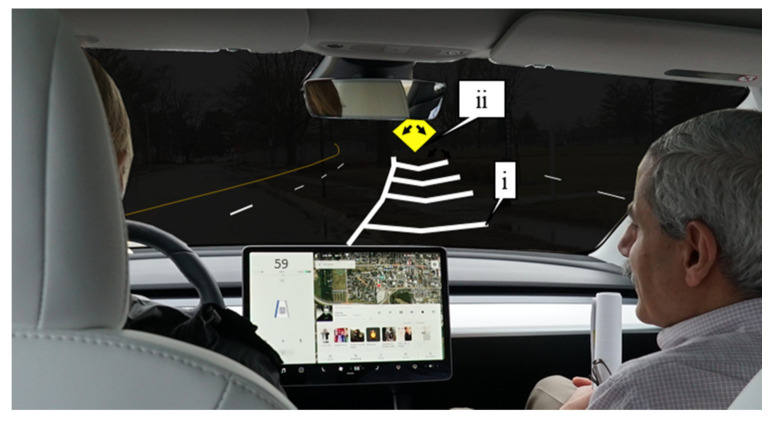
Level 2 autonomous vehicle tracking on an interstate. Available online: https://youtu.be/6QCF8tVqM3I (accessed on 1 October 2020).

Figure 3 (and video referenced in figure) shows a Level 2 autonomous vehicle losing track of the faded pavement marking on the left side of the vehicle (callout i) and heading towards a barricade (callout ii) on Interstate 94 in Chicago, Illinois [16]. As vehicle manufacturers look to increase the level of autonomy over the next decade, it is increasingly important that infrastructure is adapted and well maintained with these developments for safe operations.

## 3. Data Sets and Methods

### 3.1. Evaluation of LKA to Assess Pavement Markings on Indiana Interstates

**Figure 4:**
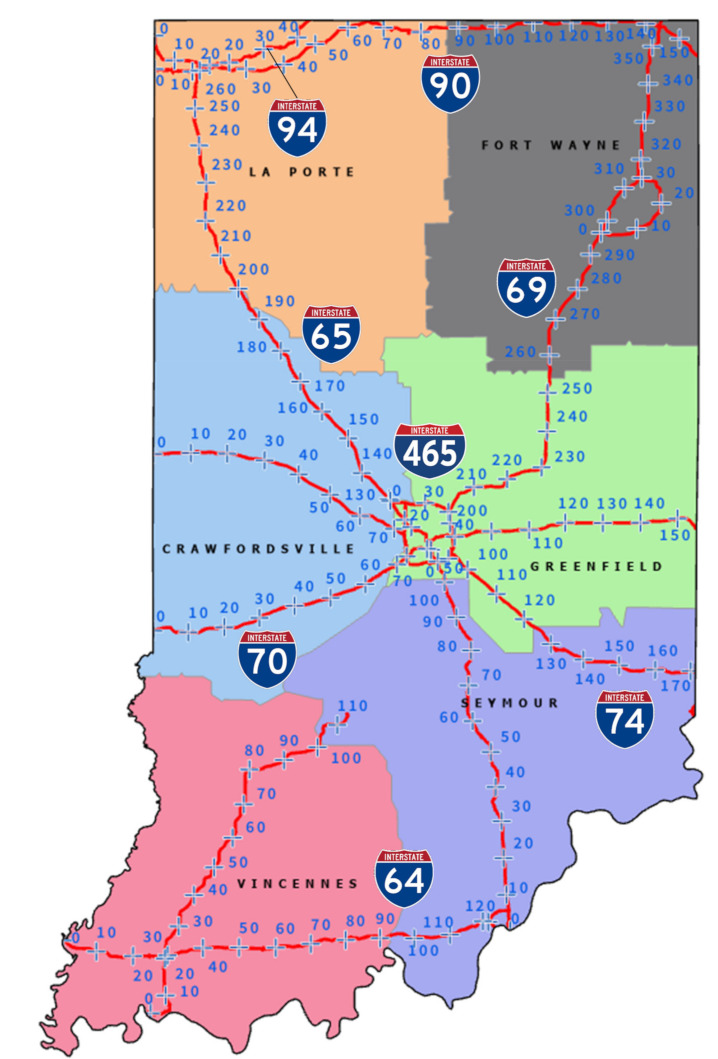
Indiana interstate system and districts.

The Indiana Department of Transportation (INDOT) has an interstate system of over 2500 miles and operates with 6 districts that manage and maintain sections of interstate within their region. Figure 4 shows the interstate mile markers and the district boundaries. This organizational structure was used when performing LKA pavement marking evaluations. This study examined the use of onboard vehicle sensors for assessing pavement markings across Indiana’s interstate system. The evaluation was a comparison of data collection between summer 2020 and winter 2021. There was considerable variation in marking visibility along the Interstate 70 (I-70) and I-65 study corridors, which is discussed further in detail.

### 3.2. Vehicle Sensor Data Collection

**Figure 5:**
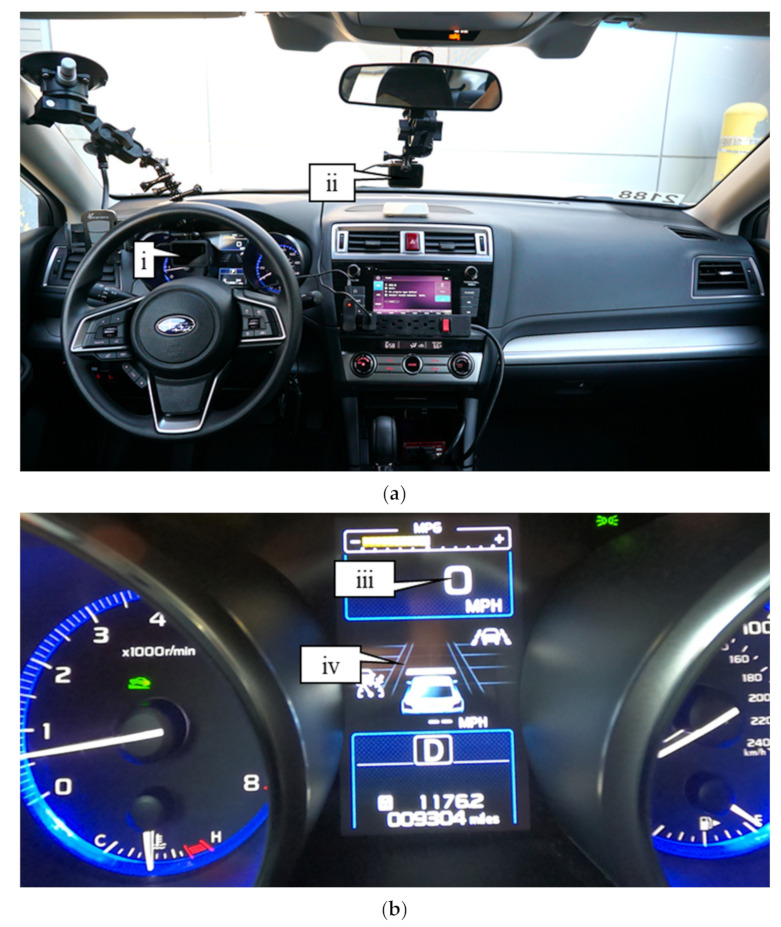
Summer 2020 data collection setup: (**a**) cab view of camera locations; (**b**) view of camera collecting instrument cluster.

To demonstrate a proof-of-concept, data were collected with two GoPro Hero 8 cameras using a University Subaru fleet vehicle during summer 2020. Figure 5a shows the data collection setup with one camera focused on the instrument cluster (callout i) of the vehicle and the other capturing the roadway conditions (callout ii). Figure 5b shows an image from the camera collecting images of the instrument cluster. The speed of the vehicle can be seen in callout iii and the LKA status in callout iv. Images were taken at half-second intervals with timestamps and GPS location information. The roadway condition images were only used for data validation.

**Figure 6:**
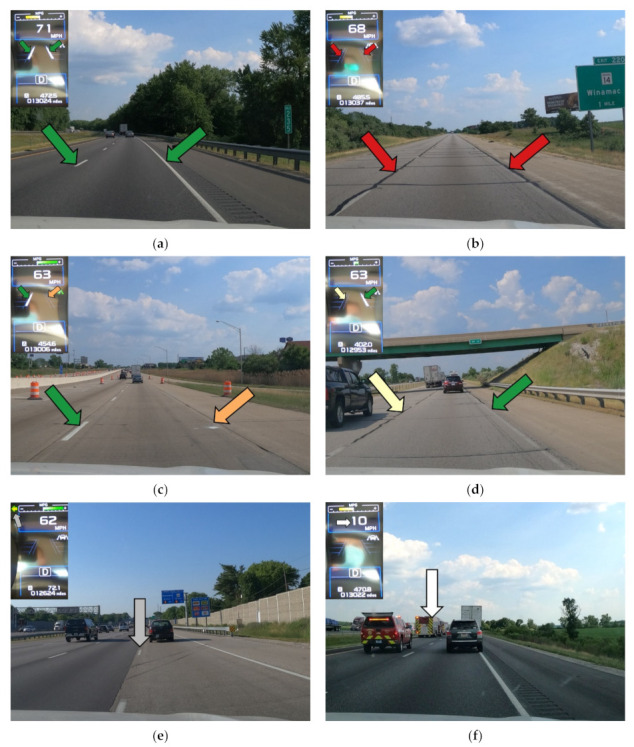
Categories of lane markings: (**a**) both detected; (**b**) both not detected; (**c**) right not detected; (**d**) left not detected; (**e**) excluded (lane change); (**f**) no data (speed less than 40 mph).

This method was used to capture images from over 2500 miles of interstate highway in Indiana over three months from June to August 2020. Over 280,000 images were taken across eight interstates and were separated into six unique categories based on the observation from the instrument cluster [17]. The vehicle performs the assessment of pavement marking detectability and provides a binary indicator (detected/not detected on the instrument cluster). The instrument cluster binary state (detected/not detected) were classified manually by a human, and the corresponding detected/not detected assessment was used in the quantitative analysis. The six categories were defined as follows:

1. Both detected: both lane markings were detected by the vehicle (Figure 6a);
2. Both not detected: Both lane markings were not detected by the vehicle with no turn signal on, and the vehicle speed was greater than 40 miles per hour (mph) (Figure 6b);
3. Right not detected: only the left lane marking was detected, but the right lane marking was not detected by the vehicle (Figure 6c);
4. Left not detected: Only the right lane marking was detected, but the left lane marking was not detected by the vehicle (Figure 6d);
5. Excluded: The turn signal was on (Figure 6e);
6. No data: The vehicle speed was less than 40 mph; 40 mph was used because the test vehicle lane-keeping feature was not active below this threshold (Figure 6f).

Subsequently, a Ford Lincoln MKZ was instrumented with a controller area network (CAN) data interface for data collection of lane marking detections to assess the quality of lane markings in February and March 2021. Discrepancies found in each data set were independently validated to ensure the difference in vehicle types did not affect the data set. The methodology was similar to the proof-of-concept completed in the summer of 2020, utilizing Go Pros to validate the observations, with the exception of no longer having to classify lane visibility using instrument cluster images but receiving the LKA signal directly from the vehicle through the CAN interface. This is particularly important to note, because the CAN bus provides a path to scaling these data collection techniques to large fleets of connected vehicles [18,19].

## 4. Results and Discussion

There were some noticeable differences between the two data collections, largely attributed to construction activities. In the summer 2020 data set, construction zones and temporary pavement markings were causing a large proportion of pavement markings to be undetected by LKA. The construction zones were not present for the winter of 2021 data collection, leading to better detection of pavement markings.

### 4.1. Indiana Interstate 65 Comparison

**Figure 7:**
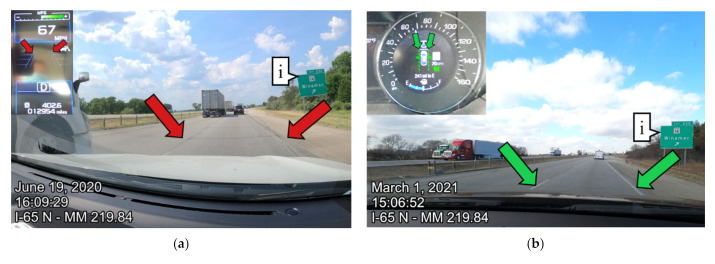

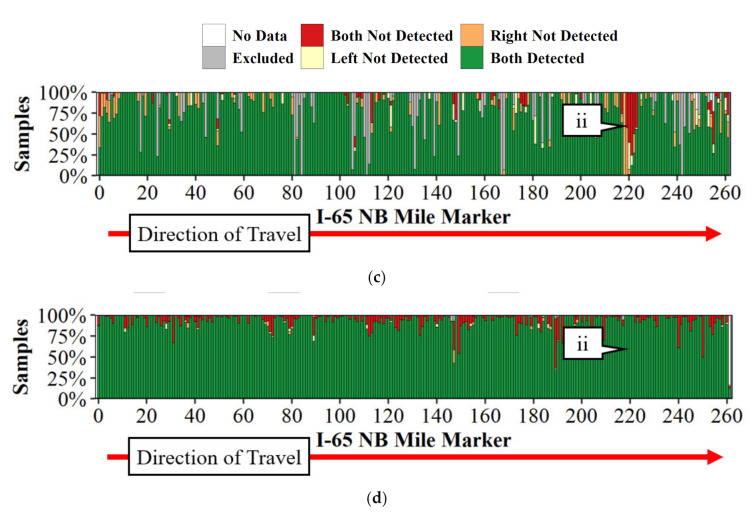
I-65 northbound 8-month lane marking comparison: (**a**) instrument and road view from I-65 northbound on 19 June 2020; (**b**) instrument and road view from I-65 northbound on 1 March 2021; (**c**) lane marking detection on I-65, 19 June 2020; (**d**) lane marking detection on I-65, 1 March 2021.

In specific locations, there were noticeable differences between the summer 2020 and winter 2021 data collections. Figure 7 shows an example of I-65 northbound from the summer 2020 data collection and winter 2021 data collection. Figure 7a shows the road view and instrument cluster in the northbound direction at mile marker 219.84. Figure 7b shows the same location eight months later when the pavement markings were repainted. Callout i is a reference sign that can be seen in both road view images. The categorizations from the instrument cluster images and the CAN interface were aggregated to the nearest tenth mile. The data were then aggregated to the nearest mile for legibility. Figure 7c shows the lane marking detection across all of I-65 northbound. The plot shows the data spatially by mile marker on the horizontal axis and the percentage of signals in each category on the vertical axis. Callout ii indicates the location where the images were taken in Figure 7a,b. During the summer of 2020, the pavement markings were not visible, while in the winter 2021 data collection, the pavement markings were repainted, making them visible to the driver as well as easily detectable by the vehicle.

### 4.2. Indiana Interstate 70 Comparison

**Figure 8:**
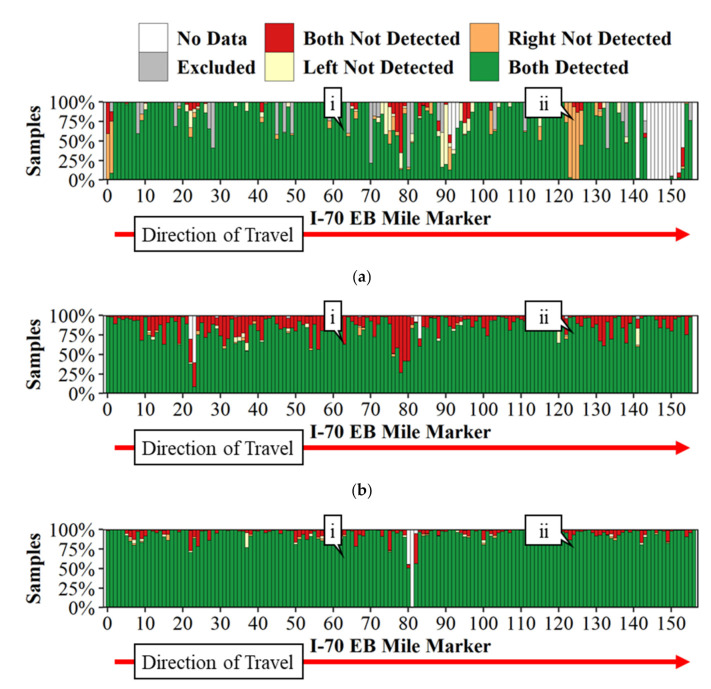
I-70 eastbound lane marking detection due to the maintenance activities: (**a**) lane marking detection on I-70, 21 July 2020; (**b**) lane marking detection on I-70, 19 February 2021; (**c**) lane marking detection on I-70, 31 March 2021.

**Figure 9:**
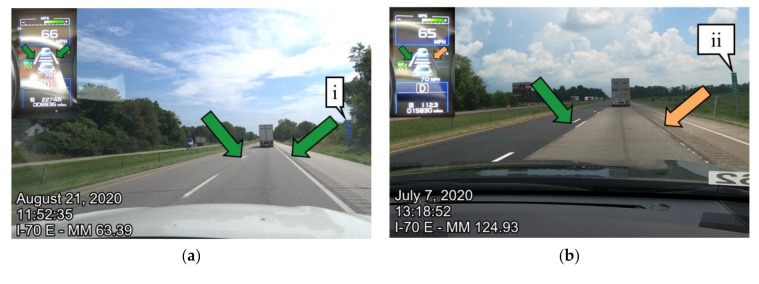

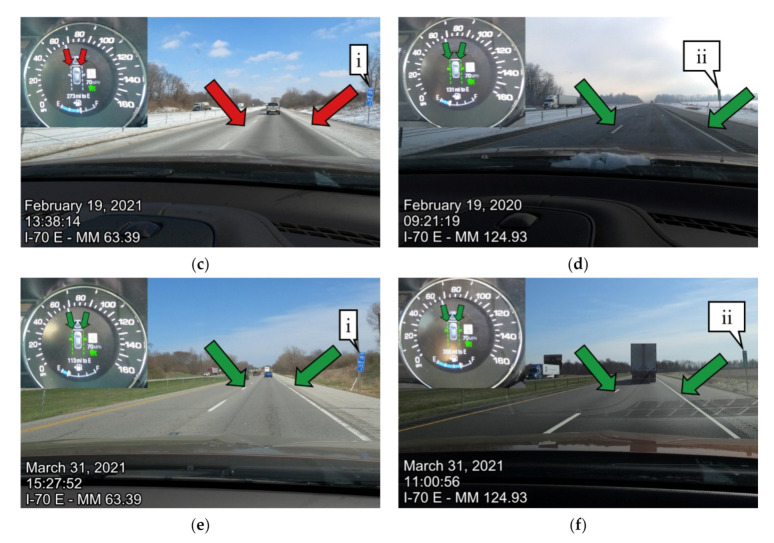
I-70 Eastbound lane marking qualitative comparisons due to the maintenance activities: (**a**) instrument and road view from I-70 eastbound milepost 63.4 on 21 July 2020; (**b**) instrument and road view from I-70 eastbound milepost 124 on 21 July 2020; (**c**) instrument and road view from I-70 eastbound milepost 63.4 on 19 February 2021; (**d**) instrument and road view from I-70 eastbound milepost 124 on 19 February 2021; (**e**) instrument and road view from I-70 eastbound milepost 63.4 on 31 March 2021; (**f**) instrument and road view from I-70 eastbound milepost 124 on 31 March 2021.

Noticeable performance differences in the detection of lane markings were observed on I-70 due to the fact of seasonal variations as shown in Figure 8. Figure 8a shows the quantitative lane detection performance across I-70 during the data collection run from summer 2020, whereas Figure 8b,c show similar performances on the same interstate during February and March, respectively, in winter 2021. Figure 9 shows example images of the road and instrument cluster for each of the data collections and at two unique points. Callout i in Figure 8 is the location of Figure 9 on I-70 eastbound at mile marker 63. During the February data collection (Figure 9c), the vehicle was unable to detect the pavement markings, which is indicated by the red both not detected arrows directing your attention to the instrument cluster and pavement markings. When comparing Figure 8a–c, it appears that Figure 8a July data show construction impact resulting in the poor lane marking detection, with limited impact due to the construction in February and March. Figure 8b has significantly more instances of both not detected that is likely due to the salt residue on the road surface. Further observation shows that salt residue on the pavement caused difficulty for the vehicle in detecting the pavement markings.

Case examples of lane marking detection with the camera image from the dash view and instrument cluster view on the same interstate I-70 eastbound are shown in Figure 9 for qualitative assessment. Callout i in Figure 8 can be seen qualitatively in Figure 9a for July 2020, Figure 9c for February 2021, and Figure 9e for March 2021. Callout i in Figure 9 references milepost 63.4, the same location as callout i in Figure 8. Similarly, lane marking detection can be seen qualitatively for callout ii in Figure 8 in Figure 9b for July 2020, Figure 9d for February 2021, and Figure 9f for the March 2021 collection. Callout ii in Figure 9 shows the vehicle approaching milepost 125.

### 4.3. Overall Interstate System Comparison

**Figure 10:**
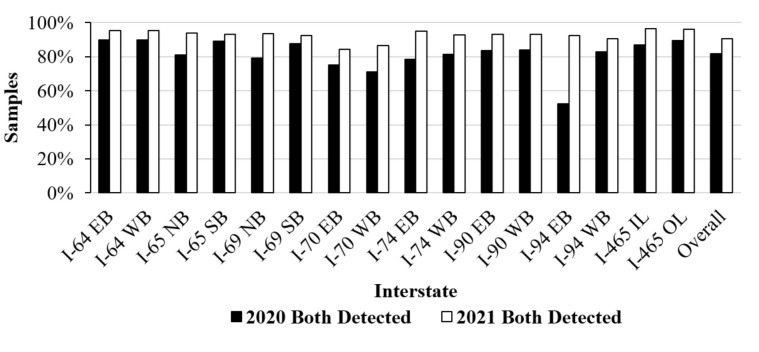
Percentage of detectable pavement markings statewide by interstate in the summer of 2020 and winter of 2021.

The results from this study can be utilized by agencies to obtain crucial information, such as pavement marking detectability, that vehicle data can provide. Not only can this information aid in preventative maintenance of the driving surface, but the data can also provide insights into lane marking detection for connected and autonomous vehicles. Figure 10 provides a summary of Indiana interstates that were detected by LKA during summer 2020 and winter 2021.

**Figure 11:**
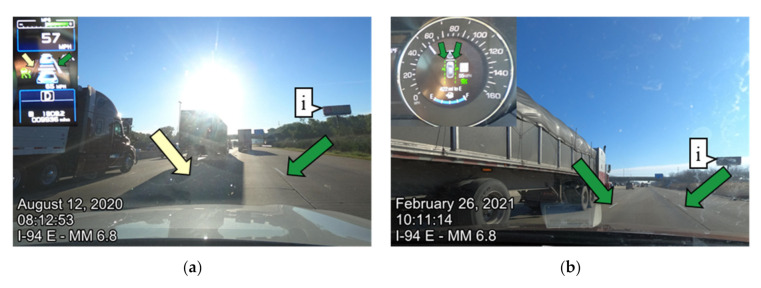

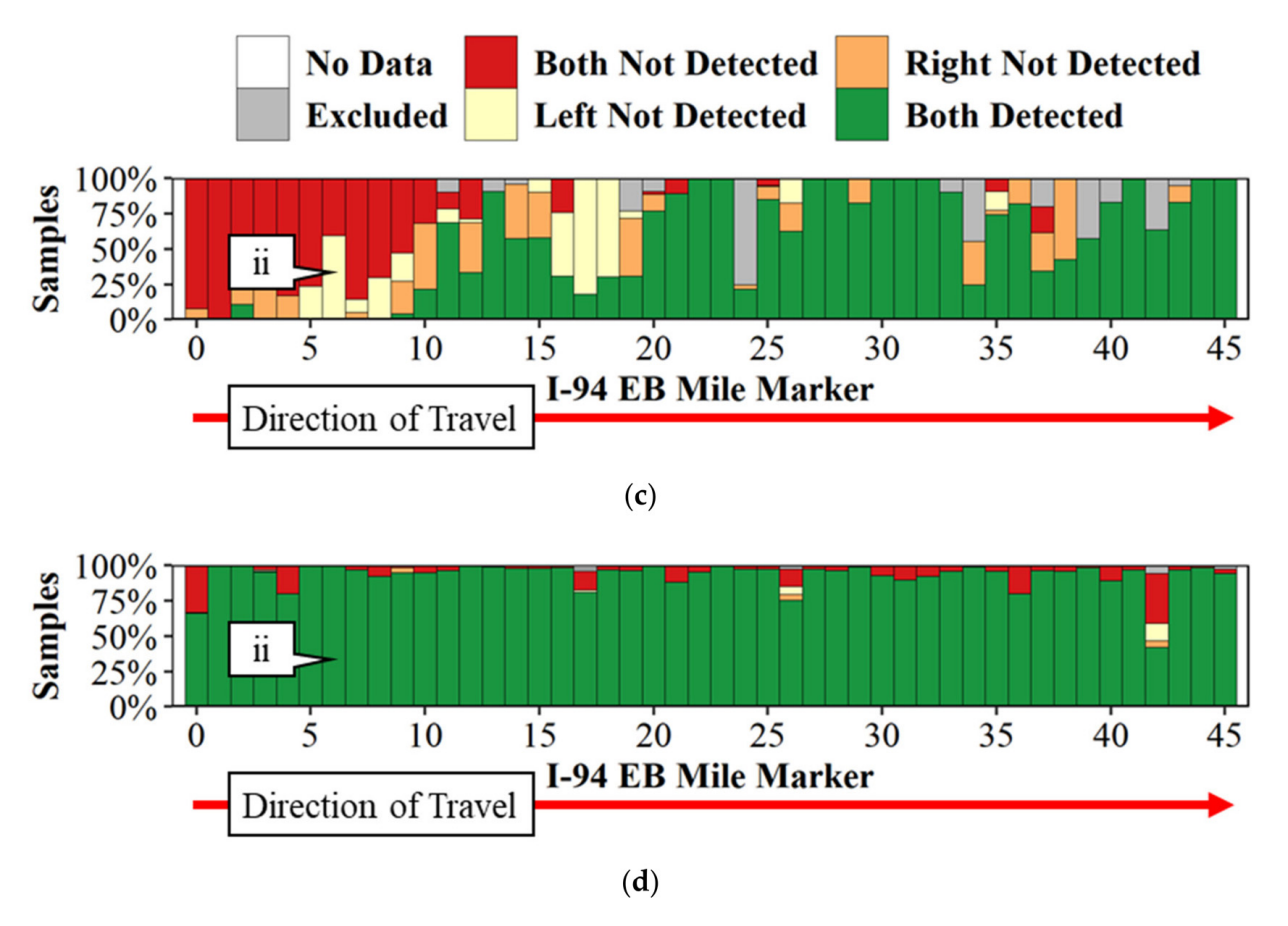
I-94 eastbound lane marking qualitative comparisons due to the fact of sun reflection: (**a**) instrument and road view from I-94 eastbound milepost 6.8 on 12 August 2020; (**b**) instrument and road view from I-94 eastbound milepost 6.8 on 26 February 2021; (**c**) lane marking detection on I-94, 12 August 2020; (**d**) lane marking detection on I-94, 26 February 2021.

During the summer of 2020, only 52.3% of lane markings were detected by LKA on I-94 eastbound (EB). This was due to the poor lighting conditions at the time of data collection. For the winter 2021 data collection, 92.6% of lane markings were detected by LKA on I-94 EB. Case examples of lane marking detection with the camera image from the dash view and instrument cluster view on the same interstate, I-94 eastbound, are shown in Figure 11 for qualitative assessment. Figure 11a shows the August 2020 data collection and qualitatively shows the impact the sun reflection has on the visibility of lane markings. Figure 11b depicts the same location during the February 2021 data collection at a different time of day where the lane markings were detected by the vehicle. Callout i in Figure 11 shows a billboard landmark for reference. Figure 11c, d show lane marking detectability for all of I-94 eastbound. Observed in Figure 11c is that a larger proportion of lane markings were not detected on the right, left, and on both sides of the vehicle from miles 0–20. The road view images suggest that the poor detection is due to the sun reflection. Figure 11d shows the February 2021, lane marking detectability and the pavement markings were detected likely because there was less impact from the sun reflection. Callout ii in Figure 11 shows the image location of Figure 11a,b.

I-70 in both directions also had fewer detected pavement markings compared to other interstates due to the lower vehicle speeds during data collection in summer 2020. The lower speeds were caused by road construction, inclement weather, and crashes. Overall LKA collected during the winter 2021 season yielded an LKA detection of 90.7% across the state of Indiana compared to summer 2020 where only 81.9% of pavement markings were both detected. The combination of construction activities, inclement weather, crashes, traffic congestion, and variable lighting conditions caused many of the detectability differences and demonstrates the importance of crowdsourcing LKA data.

Table 1 depicts the LKA lane marking detection for the Indiana interstates grouped by district. For the summer 2020 data set, a general trend can be observed; the districts with fewer miles of interstate to maintain (i.e., Crawfordsville, Fort Wayne, and Vincennes) had higher percentages of pavement markings detected. Seymour and Greenfield, the two districts with the most miles of the interstate had fewer pavement markings detected. The exception to this trend was the La Porte District, but as seen in Figure 10, I-94 eastbound had a larger proportion of pavement markings not detected due to the poor lighting and heavy traffic congestion.

**Figure 12:**
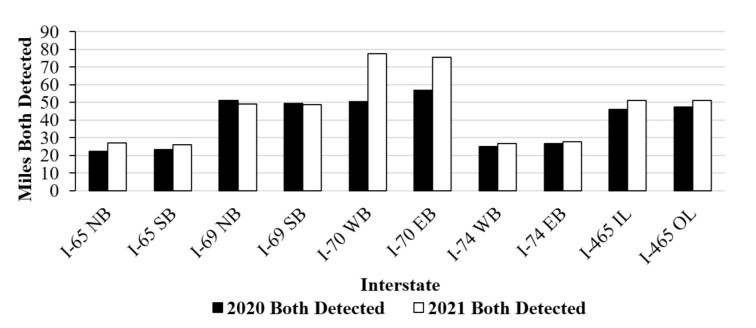
Miles of interstate detected by LKA in the Greenfield District in summer 2020 and winter 2021.

Figure 12 shows LKA data specifically for Greenfield District. This plot shows the interstate and direction on the *x*-axis and the miles of the detected interstate on the *y*-axis. Greenfield manages 29 miles of I-65, 55 miles of I-69, 88 miles of I-70, 50 miles of I-74, and 53 miles of I-465. The trends observed when looking across all interstates can also be observed when looking at the sections of interstate that lie in Greenfield. Overall, fewer pavement markings were detected in the summer 2020 data collection than the winter 2021 data collection. Significant differences in the number of miles with both detected lane markings were observed for I-70 in Greenfield during the two data collection periods. When considering both directions on all interstates, this comes to a total of 550 miles of the interstate for the district to manage. The interstate with the largest difference in detectability was I-70, where in summer 2020 less than 65% of pavement markings were detected. This was due to the presence of three large construction projects on the interstate over the summer of 2020 causing lower vehicle speeds and some of the pavement markings to not be detected.

## 5. Conclusions

Determining locations where both a human driver and autonomous vehicle cannot detect pavement markings is and will continue to be important for transportation agencies and original equipment manufacturers. This study explored the use of vehicle lane keep assist (LKA) systems to detect and determine pavement marking conditions. Several use cases were examined for validation purposes on I-65 and I-70. The results showed that LKA can be utilized in identifying detectible pavement markings under various conditions and that it has potential use by transportation agencies to efficiently gather pavement marking evaluation over large road networks. Several factors that affect LKA detection must be accounted for including sunlight, weather, and vehicle speeds. Information from the winter 2021 data set can be used to prioritize maintenance activities for restriping the remaining 9.3% of pavement markings not detected by the vehicle. Further development for implementation should be considered to provide more frequent and accurate measurements.

### Future Opportunities with Real-Time Lane Marking Quality Dashboards

On-board vehicle sensors provide a robust system for agencies to collect quantitative data regarding pavement marking visibility. Although a single vehicle could be driven by an agency around the state to replicate the standard practice with specialized vans, a much more scalable approach would be to systematically collect this data from a sample or an entire fleet of vehicles. Such an approach would allow an agency to have a dynamic, crowdsourced view of their lane marking detection in a variety of conditions, such as sunny, cloudy, wet pavement, heavy rain, light snow, and salt coverage, as well as to track how these lane markings deteriorate over time.

The methodology for inventorying lane marking data for one vehicle can be scaled using cellular network connectivity and cloud infrastructure. The data could be fed through a CAN interface and buffered and aggregated over predetermined distance or time intervals to keep the data size small. Only the pertinent properties, such as locations with over-threshold or interesting markings, would be kept. The data can be batched in either daily or minute batches before uploading over the cellular connection to a data warehouse. A dashboard, reporting system, or user interface can then be built on top of an analysis engine table to present the information to authorities or stakeholders. To ensure an accurate data set, it would be important to obtain data from a diverse fleet due to the varying performances of vehicle sensors.

**Figure 13:**
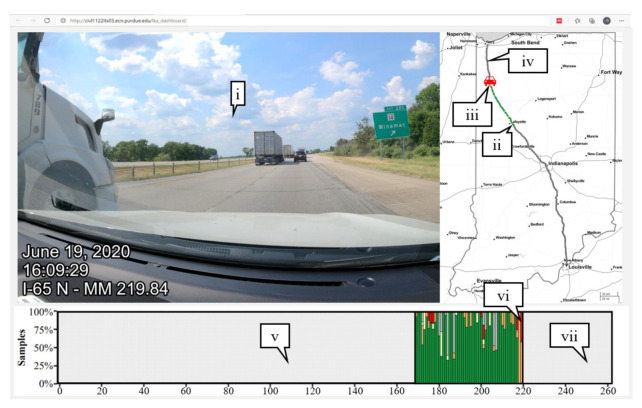
Pilot application demonstrating near-real-time data collection.

Figure 13 shows an example of how such an interactive user interface would look. The web application would connect to a cloud database system to retrieve dashboard imagery of opted-in drivers or fleet vehicles at a specific location (callout i), a map of the route travelled by a vehicle (callout ii), and the current location of a vehicle (callout iii). The database would also have knowledge of the total route to be travelled (callout iv). The route is also linearized by mile in the bottom graphic (callout v). As the vehicle collects data, the road conditions are categorized, and each bar would be populated according to the distribution of this and other vehicles (callout vi). Any locations that this vehicle or collection of vehicles have not yet travelled would be shown in grey (callout vii).

## Figures and Tables

**Table 1:** Lane marking detection by district.

District	Total Miles of Interstate	2020 Percent Both Detected	2021 Percent Both Detected
Crawfordsville	426	84.9%	89.8%
Fort Wayne	342	88.1%	90.8%
Greenfield	550	75.9%	90.3%
La Porte	388	75.2%	92.6%
Seymour	554	73.7%	94.2%
Vincennes	392	88.4%	96.3%
All Districts	2652	80.2%	92.3%

## Data Availability

Not applicable.
